# Sterilization of Exopolysaccharides Produced by Deep-Sea Bacteria: Impact on Their Stability and Degradation

**DOI:** 10.3390/md9020224

**Published:** 2011-02-10

**Authors:** Emilie Rederstorff, Ahmed Fatimi, Corinne Sinquin, Jacqueline Ratiskol, Christophe Merceron, Claire Vinatier, Pierre Weiss, Sylvia Colliec-Jouault

**Affiliations:** 1 Laboratory of Biotechnology and Marine Molecules, French Research Institute for Exploitation of the Sea (IFREMER), Rue de l’Ile d’Yeu, BP 21105, 44311 Nantes Cedex 03, France; Email: Emilie.Rederstorff@ifremer.fr (E.R.); Corinne.Sinquin@ifremer.fr (C.S.); Jacqueline.Ratiskol@ifremer.fr (J.R.); 2 INSERM UMRS 791, Laboratory of Osteo-Articular and Dental Tissue Engineering, School of Dental Surgery, University of Nantes, 1 Place Alexis Ricordeau, 44042 Nantes Cedex 1, France; Email: ahmed.fatimi@univ-nantes.fr (A.F.); christophe.merceron@univ-nantes.fr (C.M.); claire.vinatier@sante.univ-nantes.fr (C.V.); pweiss@sante.univ-nantes.fr (P.W.)

**Keywords:** marine biotechnology, polysaccharides, sterilization procedures, characterization, molecular weight distribution, rheology

## Abstract

Polysaccharides are highly heat-sensitive macromolecules, so high temperature treatments are greatly destructive and cause considerable damage, such as a great decrease in both viscosity and molecular weight of the polymer. The technical feasibility of the production of exopolysaccharides by deep-sea bacteria *Vibrio diabolicus* and *Alteromonas infernus* was previously demonstrated using a bioproduct manufacturing process. The objective of this study was to determine which sterilization method, other than heat sterilization, was the most appropriate for these marine exopolysaccharides and was in accordance with bioprocess engineering requirements. Chemical sterilization using low-temperature ethylene oxide and a mixture of ionized gases (plasmas) was compared to the sterilization methods using gamma and beta radiations. The changes to both the physical and chemical properties of the sterilized exopolysaccharides were analyzed. The use of ethylene oxide can be recommended for the sterilization of polysaccharides as a weak effect on both rheological and structural properties was observed. This low-temperature gas sterilizing process is very efficient, giving a good Sterility Assurance Level (SAL), and is also well suited to large-scale compound manufacturing in the pharmaceutical industry.

## 1. Introduction

Carbohydrates, particularly those found on cell surfaces, play critical biological roles, such as glycoconjugates or proteoglycans and especially their polysaccharidic chains called glycosaminoglycans. The discovery of the biological importance of these carbohydrates marked the beginning of glycobiology, glycomics, carbohydrate-based drug discovery and glycomimetic drug development [1,2]. Bacterial exopolysaccharides (EPS) serve distinct biological functions; one of the more important functions of EPS involves adhesion of cells to natural and artificial surfaces. EPS immobilize biofilm cells and keep them in close proximity, thus allowing for intense interactions, including cell-cell communication [3,4,5]. 

The recent development of powerful bacterial engineering tools, such as large-scale fermenters, allows the production of bacterial polysaccharides at a viable economic cost. Consequently the replacement of polysaccharides of mammalian or plant origin by microbial polysaccharides produced by biotechnological processes (such as alginate, heparin precursor, hyaluronic acid, *etc.*) is possible [6,7,8]. The marine realm is a rich and largely untapped resource of products that are of potential interest for drug discovery [9,10,11,12,13]. Biopolymers from marine prokaryotes offer significant structural diversity with novel material and biological properties. Biologically active glycopolymers derive their action from their molecular structure; including molecular size, polydispersity and repeating unit features varying in size, stucture, linkages, and carried substituents. When sulfated, carbohydrates may be glycosaminoglycan-like components that exhibit many interesting properties with potential medical applications [14,15].

**Figure 1:**
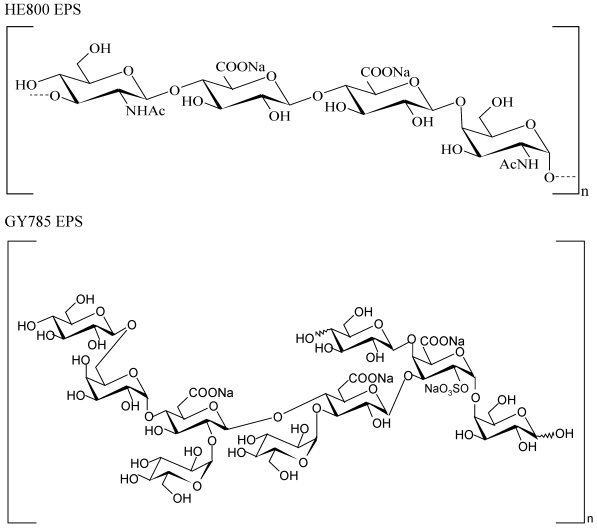
Repeating units of marine bacterial polysaccharides produced by *Vibrio diabolicus* (HE800 EPS) and *Alteromonas infernus* (GY785 EPS), respectively.

Deep-sea hydrothermal vents discovered 30 years ago are a new source of a wide variety of fascinating microorganisms that are well-adapted to these extreme environments [16]. The screening of a large number of isolates obtained from different oceanographic cruises led to the discovery of new species able to produce unusual EPS [17]. Among them, the first species of *Vibrio* to be isolated from such an extreme environment was a mesophile *Vibrio diabolicus,* that secretes a linear EPS with a tetrasaccharide repeating unit, showing some resemblance to hyaluronic acid (Figure 1) [18,19]. This high-molecular-weight HE800 EPS (8 × 10^5^ g/mol) secreted by *Vibrio diabolicus* exhibits interesting biological activities useful for increasing bone formation. HE800 EPS was evaluated on the restoration of bone integrity in an experimental animal model and was demonstrated to be a strong bone-healing substance without inducing any inflammatory reaction [20]. Another new mesophilic species, *Alteromonas infernus*, produces a branched high-molecular-weight GY785 EPS (1.5 × 10^6^ g/mol) with a nonasaccharide repeating unit (Figure 1) [21,22]. Low-molecular-weight oversulfated derivatives from GY785 EPS were isolated in order to obtain heparin-like compounds. These derivatives had anticoagulant [23] and proangiogenic properties [24].

The aim of this study is to select an appropriate sterilization method, which is a crucial step for biomedical purposes, to improve the biotechnological processing of these two marine bacterial EPSs. For many natural products, downstream processing involves several critical steps such as isolation, purification, sterilization and drying, *etc.* The yields of these two EPSs are relatively high, ranging from 1 to 6 g/L of culture medium. They both can be produced on an industrial scale with very good yields and competitive total costs. As the sterilization process may affect the physical and chemical properties of the product and also induce loss of biological activity, finding suitable sterilization methods remains a challenge, especially for both heat and moisture sensitive molecules such as polysaccharides. The destructive effect of heating on polysaccharides is well described [25,26]. Therefore in this study, we selected other sterilization methods such as ethylene oxide or EO, radiation by gamma and beta rays and cold plasmas, in order to evaluate the most appropriate sterilization procedure for the linear HE800 and branched GY785 EPSs. 

Chemical sterilization by EO or cold plasma is effective in killing or eliminating a wide range of pathogens (such as fungi, bacteria, viruses, spore forms, *etc.*) and provides a good sterility assurance level (SAL set at 10^−6^ means there is a one-in-a-million chance that a live microbe is in the sterilized material). The use of gas or liquid chemical sterilizers avoids the problem of heat damage but often this can present some health and environmental risks [27]. Sterilization methods using radiation, such as gamma and beta rays, are very good methods for achieving a high SAL. In many cases, the required SAL can be achieved by using a uniform treatment dose of 25 kGy (2.5 Mrad), as recommended by pharmacopoeia [28]. The moderate use of EO is due to its well-known health effects. Strict regulations have been established concerning EO sterilization. Hospitals nevertheless choose this method because they can get the best possible SAL with EO and high throughput.

## 2. Results and Discussion

### 2.1. The Rheological and Viscous Properties of Polysaccharides

**Figure 2:**
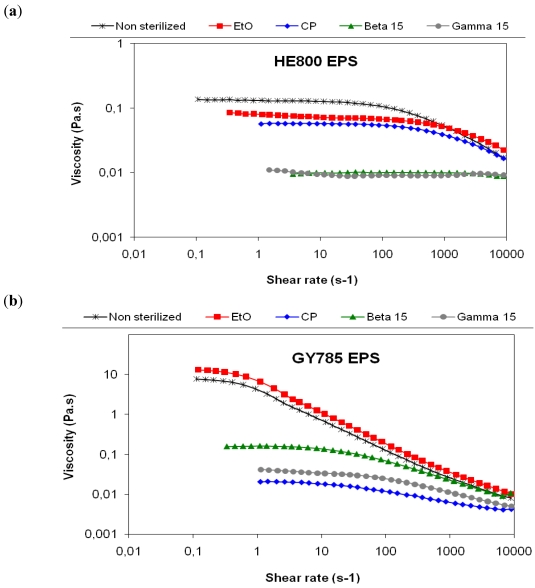
Flow curves of the untreated (control) and treated EPS in water solutions at 25 °C. (**a**) HE800 EPS produced by *Vibrio diabolicus* (1.5% w/w); (**b**) GY785 EPS produced by *Alteromonas infernus* (1.25% w/w).

The rheological measurements show non Newtonian pseudoplastic behavior of both non sterilized HE800 and GY785 polymer solutions (Figure 2). The EO sterilization decreased slightly the HE800 EPS viscosity and did not affect the rheological properties of the GY785 EPS. For the untreated HE800 EPS solution, the limiting Newtonian viscosity *η*_0_ was 0.14 and after sterilization treatments 0.08, 0.06, 0.01 and 0.01 Pa·s for the EO, cold plasma (CP), beta and gamma sterilizations, respectively. The limiting Newtonian viscosity *η*_0_ calculated with the cross equation showed 8 Pa·s for the non sterilized GY785 EPS, 15 Pa·s after EO sterilization, 0.2, 0.04 and 0.03 Pa·s after beta, gamma and CP sterilizations, respectively.

For the HE800 EPS solution, EO and CP sterilizations decreased the viscosity from about one order of magnitude and maintained the pseudoplastic typical shape of polymer curves. With beta and gamma irradiations at a dose of 15 kGy (1.5 Mrad), the viscosity decreased dramatically with a Newtonian behavior characteristic of water or small molecules in water solution.

The EO sterilization of GY785 EPS increased the *η*_0_ twofold but it maintained the same order of magnitude. EO probably interacts with the branched macromolecule even if the residual amount of EO after desorption is very low (10 ppm), but the curve was the same as that obtained for untreated EPS and in a logarithmic scale both untreated and treated EPSs showed very similar behavior. Surprisingly, no increase was observed for the linear HE800 EPS whereas the residual amount of EO after desorption was higher (30 ppm). No data were found in literature to explain this unpredictable effect on the viscosity. Clearly, a Newtonian plateau observed at low shear rates is followed by a shear-thinning behavior for the non sterilized macromolecules [29]. At high shear rate the viscosity shows a power-law dependence with the shear rate. After beta sterilization at a dose of 15 kGy (1.5 Mrad), GY785 EPS is still a macromolecule because the curve shows two parts. For the first part, which is under 10 s^−1^, the viscosity remains constant with an increase of the shear rate. After a critical shear rate, there is always a decrease of the viscosity due to alignment of the macromolecules in the flow. The slope of this second part of the curve is less pronounced than the second part of the other curves obtained for non sterilized GY785 EPS and EO sterilized GY785 EPS (Figure 2b). This pseudoplastic behavior is very common in the case of macromolecular solutions [30,31]. It is less pronounced after beta sterilization than the non-sterilized or EO sterilized macromolecules with a decrease of the value and the extent of the Newtonian plate, under 10 s^−1^. These flow modifications suggest a partial degradation of the macromolecules, but not complete [32]. In the cases of CP and gamma sterilizations, *η*_0_ were very low with a decrease of about three orders of magnitude for GY785 EPS, and showed a linear curve of Newtonian solution characteristic of small molecules in solution. GY785 EPS was highly degraded by these two sterilization procedures.

This rheological data suggests that irradiation, even at a dose of 15 kGy (1.5 Mrad), is not a good method for the sterilization of HE800 and GY785 polysaccharides tested. CP can be used for sterilization of HE800 EPS based on its slight loss of viscosity in comparison to the large loss of viscosity for GY785 EPS.

### 2.2. Molecular Weight and Polydispersity of Polysaccharides

**Figure 3:**
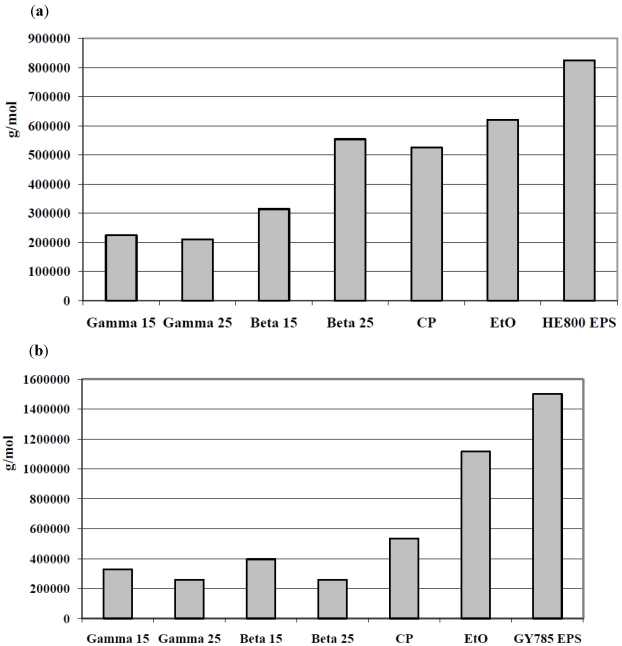
Weight-average molecular weight (Mw) of the untreated (control) and treated EPSs. (**a**) HE800 EPS produced by *Vibrio diabolicus*; (**b**) GY785 EPS produced by *Alteromonas infernus*.

The weight-average molecular weight (Mw) was determined for each treated EPS sample and compared to untreated (or native) EPSs (8 × 10^5^ and 1.5 × 10^6^ g/mol for HE800 and GY785 EPSs, respectively). All sterilization methods induced a decrease in the weight-average molecular weight compared to native products (Figure 3). Irradiation methods and especially gamma rays induced the highest decrease in both EPSs (<2.5 × 10^5^ g/mol). A lesser decrease was observed in cold plasma treatment, but the weakest impact on molecular weight was obtained when the EPSs were treated by EO. In the case of the linear HE800 EPS, a molecular weight of 6.5 × 10^5^ compared to 8 × 10^5^ was obtained and with the highly branched GY785 EPS, 1.1 × 10^6^ compared to 1.5 × 10^6^ g/mol. The advantage of a multi-angle laser light scattering (MALLS) detection consists in an additional possibility to determine dimensions of dissolved polymer in terms of gyration radius Rg from the angular dependence of scattered light. A decrease of the radius of gyration Rg was also obtained in treated EPSs, irradiation treatment gave the greatest decrease (<40 nm compared to 55 and 112 nm for native HE800 and GY785 EPSs, respectively). This data could suggest a discrepancy between the weak effect observed on rheological properties and the reduction in molecular weights for both EPSs, especially in the case of the EO treatment. This discrepancy could be explained by the concentrations of the EPSs in the rheological measurement and molecular weight determination methods (>1% w/w and 0.2% w/w, respectively) as they are not of the same range. In a concentrated polymer solution, both intra- and intermolecular interactions are higher than in diluted polymer solution [32]. EO sterilization had a weaker impact than the other sterilization methods on the radius of gyration Rg, 44 and 86 nm for the treated HE800 and GY785 EPSs, respectively. Our data show that there is a molecular weight dependence of the radius of gyration. Because the Rg is related to the volume occupied by the molecule in a solution, the relationship between Rg and Mw suggests that the untreated and treated polysaccharides have similar structures or patterns [33].

**Figure 4:**
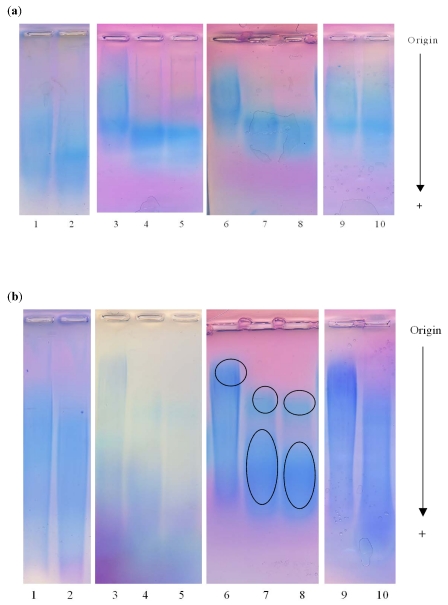
Electroforetic migration of the untreated (control) and treated EPSs: **1**: Control; **2**: EO; **3**: Control; **4**: Gamma 15; **5**: Gamma 25; **6**: Control; **7**: Beta 15; **8**: Beta 25; **9**: Control; **10**: CP. (**a**) HE800 EPS produced by *Vibrio diabolicus*; (**b**) GY785 EPS produced by *Alteromonas infernus*.

These results were confirmed by the electrophoretic patterns of treated EPSs (Figure 4). This methodology was not used to determine the molecular weight of the polysaccharides but to observe the changes in the dispersity of chain sizes for the treated polysaccharides compared to the untreated ones. The molecular weight was previously determined by SEC/MALLS analyses. As often observed for high molecular weight polysaccharides, contrary to proteins for which distinct bands are obtained, the polysaccharides separate into smears and consequently it is difficult to obtain a good staining intensity. All EPSs treated with gamma and beta rays presented a narrower pattern with greater mobility than the native product, and especially for linear HE800 EPS. GY785 EPS treated with beta rays presented two distinct spots with different mobilities. The spot with greater mobility represented the majority of the polysaccharidic chains. The migration patterns of EPSs treated with EO were very similar to their respective native EPSs, showing a broad smear suggesting a polydisperse EPSs.

The decrease in the molecular weight of some polymers and polysaccharides is well described. At increased temperature, even in the absence of water, polymer degradation occurred [34]. Water soluble polysaccharides are very sensitive to chemical and physical parameters. Degradation mechanisms include acidic hydrolysis, oxidative reductive depolymerisation (ORD) and β-elimination. Only at room temperature and in a dry place, polysaccharides are stable and preserved against depolymerization and storage [26,34]. Contrary to the conventional methods such as dry or moist heat sterilization that cause degradation and hydrolysis, radiation sterilization such as beta and gamma rays have become a very popular procedure. Nevertheless, if irradiation sterilization has been the method of choice for medical devices and gives very good results for food applications (spices, ground meats, *etc.*); this recent method has some limitations especially on therapeutic agents and polymers. The radiation generally interacts with polymers. Two major mechanisms of degradation occur: chain scission which reduces the molecular weight and some rearrangement of network structure or cross-linking, which results in the formation of large three-dimensional networks. In the case of the bacterial EPSs examined in this study, the first mechanism occurs in the same way as described for hyaluronic acid in which glycosidic cleavage is the most widely observed consequence of radiation action on it [35,36,37].

Gas plasma technology is an alternative sterilization method to circumvent the limitations of traditional instruments. It is a new method for the sterilization of heat-sensitive substances. The plasmas used for sterilization are ionized gases (O_2_, N_2_, H_2_, air, *etc.*).UV photons emitted by excited species produce the direct destruction of a wide range of microorganisms by UV irradiation and reactive species, but they can induce chemical reactions [27,28,29,30,31,32,33,34,35,36,37,38]. Some recent studies describe the effect of gas plasma sterilization on polymers. In the case of polyethylene, no changes in chemical composition of the polymer due to the hydrogen peroxide sterilization process were observed [39]. On the other hand, exposure to a gas plasma sterilization process using hydrogen peroxide resulted in significant degradation in based polyurethane elastomer [40]. In our experiment, a mixture of gases composed of O_2_ and N_2_ was used to sterilize EPSs. Both EPSs were severely depolymerized by this cold plasma treatment.

Like gas plasma, EO gas is commonly used to sterilize objects sensitive to temperatures greater than 60 °C, such as plastics. EO treatment is generally carried out between 30-60 °C with relative humidity above 30%. This is a simple method and is very convenient for sterilization on an industrial scale. Marreco *et al.* [41] compared different sterilization methods and showed that EO can be considered the most adequate sterilizing agent for chitosan membranes as it preserves morphology, mechanical properties and cytotoxicity of this kind of membrane. In 2002, Barbucci *et al.* [42] also evaluated different sterilization methods for hyaluronic acid hydrogel used in the treatment of osteoarthritis. They noted that EO and gamma rays do not modify the characteristics of the hydrogel, in terms of swellability nor destroy the network structure of the hydrogel. On the contrary, gamma ray sterilization induces breakage of this linear polysaccharide. The glycosidic cleavage described for hyaluronic acid is also observed for both HE800 and GY785 EPSs. The most appropriate method in this study seems to be EO sterilization which causes less damage in terms of molecular weight reduction than the other methods tested.

### 2.3. Chemical Characterization

**Figure 5:**
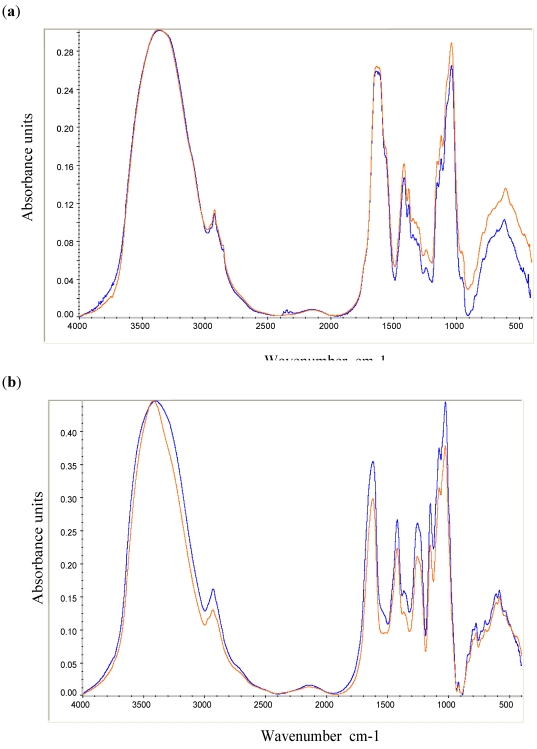
FTIR spectra of the untreated (control) and treated EPSs by gamma rays at a dose of 15 kGy: control (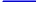) and gamma (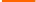). (**a**) HE800 EPS produced by *Vibrio diabolicus*; (**b**) GY785 EPSproduced by *Alteromonas infernus*.

The Fourier transform infrared spectroscopy (FTIR) analysis was applied to detect chemical modifications on treated samples. The FTIR spectra did not show important chemical modifications on all treated samples, even on irradiated samples (Figure 5). In Figure 5a, the FTIR spectra of the native and gamma ray treated HE800 EPS were very similar and no difference was observed in the most representative signals. Both spectra exhibited a broad O-H stretching band at 3400-3200 cm^−1^, a minor C-H stretching band at 2900 cm^−1^, a large absorption band at 1630-1650 cm^−1^, an intensive absorption band at 1050 cm^−1^ assigned to the C-O and C-O-H vibrations and a specific broad absorption band at 1550 cm^−1^ associated with N-H stretching vibration. In Figure 5b, a few differences were observed between the native and gamma ray treated GY785 EPS. The spectrum of the treated EPS exhibited an O-H stretching band less broad than the native EPS; a weak shoulder observed at 1550 cm^−1^ for the native EPS was not present in the irradiated EPS. The well-known carbohydrate bands between 1000 and 1200 cm^−1^ were found in both spectra as well as the ester sulfate bands at 1250 cm^−1^ and carboxylate bands at 1450 and 1610 cm^−1^.

Using this spectroscopic technique, no significant chemical modifications were observed in all treated samples. Very little data was found in the literature on FTIR analysis used to detect chemical modifications to products after sterilization, especially in polysaccharides. This analysis can be applied to detect oxidation or cross-linking processes [43]. The oxidation of ultra-high molecular weight polyethylene after supercritical CO_2_ sterilization was analyzed by this spectroscopic technique, and no effect on the chemical integrity of this material was detected [44]. In our study none of these processes seems to have occurred, suggesting only a structural modification to the size or molecular weight of the polysaccharidic chains.

### 2.4. Sterility of Polysaccharides

For all the different types of sterilization methods tested, after 48 h of incubation on PCA medium at 37 °C, the number of total bacteria/g of treated EPS was zero, compared to 2 × 10^6^ and 10^3^ total bacteria/g for untreated HE800 and GY785 EPSs, respectively (data not shown). According to the results obtained, the two treated EPSs were effectively sterilized by all sterilization methods used (ethylene oxide, radiation by gamma and beta rays and cold plasma).

### 2.5. Toxicity of Polysaccharides

**Figure 6:**
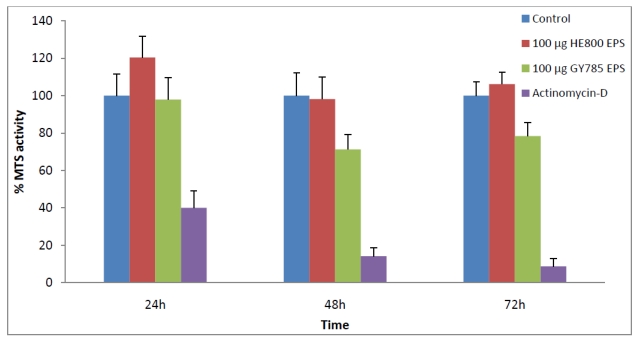
Cytotoxicity of the HE800 and GY785 EPSs treated by ethylene oxide. Cells were cultured during 24, 48 and 72 h without (control) or with 100 µg of treated EPS or with Actinomycin-D (control for cytotoxicity) per well. Values are mean ± SD of 4 experiments.

The Immediately Dangerous to Life and Health level (IDLH) for EO is 800 ppm. The concentration found in both HE800 and GY785 EPSs was below 30 ppm. This concentration can be further reduced by increasing the desorption time or the period of post-sterilization aeration to remove toxic residues. The cytotoxicity of the EPSs treated with EO was evaluated on primary articular cartilage cells (Figure 6). In our experiment, neither EPSs treated with EO showed any cytotoxicity to the cells at the concentration used.

## 3. Experimental

### 3.1. Materials

HE800 and GY785 exopolysaccharides (EPS) were purchased from Seadev-FermenSys SAS (France). HE800 and GY785 EPSs were obtained from the deep-sea, mesophilic, aerobic, and heterotrophic bacteria *Vibrio diabolicus*, *Alteromonas infernus*, respectively. For the meaning of the EPS names, the letters HE and GY are the abbreviation of the name of the oceanic cruises: HERO and GUAYNAUT, respectively. The meaning of the numbers is the sample number given during the cruise. The isolation procedure and characteristics of the HE800 *Vibrio diabolicus* and GY785 *Alteromonas infernus* strains and also the production, purification and characterization of the HE800 and GY785 EPSs were previously described [18,19,20,21]. The purities of the final products (HE800 EPS and GY785 EPS) were estimated to be >80%, they are hygroscopic so they may contain in dry state from 10 to 20% of water (w/w) and are mainly in sodium salt form (Na: 4%; Ca: 1%) [45,46].

### 3.2. Sterilization Procedures

Different types of sterilization methods were tested for polysaccharides. These include widely-used sterilization methods, such as radiation and ethylene oxide, as well as less conventional methods, such as gas plasmas.

#### 3.2.1. Ethylene Oxide

Ethylene oxide sterilization was performed by Stérylène, Groupe IONISOS (Gien, France) on lyophilized polysaccharides. The samples were exposed to ethylene oxide with a concentration of 700 mg/L for 250 min at 50 mbar pressure and a temperature of 45 °C with a relative humidity of 45% followed by desorption treatment in order to remove the residual ethylene oxide. After treatment, the amount of residual ethylene oxide was quantified for each polysaccharide by Stérylène.

#### 3.2.2. Radiation

Sterilization using radiation by gamma rays (with cobalt-60 as source) and beta rays (by electron beam) was performed at different doses (15 and 25 kGy) on lyophilized polysaccharides by Groupe IONISOS, Sablé-sur-Sarthe and Orsay (France), respectively. The dose of 25 kGy (2.5 Mrad) is usually recommended by pharmacopoeia and the dose of 15 kGy (1.5 Mrad) was tested to check whether the sterilization was sufficient without inducing important degradation of the EPSs.

#### 3.2.3. Cold Plasmas

The cold plasma sterilization was done by CRITT MDTS, Charleville-Mézières (France). The lyophilized polysaccharides were sterilized using a mixture of ionized gases (plasmas) composed of O_2_ (20%) and N_2_ (80%) for 1 h.

### 3.3. Characterization of Marine Polysaccharides

#### 3.3.1. Steady Shear and Dynamic Oscillatory Measurements

Rheological measurements were performed using the Rheo Stress 300 rheometer (ThermoHaake^®^, Germany) with titanium cone-plate geometry (60 mm diameter, 1° cone angle, 52 µm gap). Steady shear tests were carried out at 25 °C on different polymer solutions according the studied polymer (1.5% w/v for HE800 EPS and 1.25% w/v for GY785 EPS) before and after sterilization. The operating shear rate ranged from 0.1 to 9000 s^−1^. Different flow curves were fitted and extrapolated to lower shear rates by the Cross equation [47].


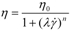
      (1)

where *η* (Pa.s) is the viscosity at a given shear rate 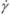 (s^−1^); *λ* is the structural relaxation time (s); *n* is the exponent in the power law regime and *η_0_* (Pa.s) is the zero shear viscosity (*i.e.*, limiting Newtonian viscosity).

#### 3.3.2. Molecular Weight by SEC/MALLS Analyses

Polysaccharides were dissolved in distilled water at a concentration of 2 mg/mL (0.2% w/v) and filtered on 0.45 µm cellulose acetate syringe filter. The weight-average molecular weight (Mw), number-average molecular weight (Mn), radius of gyration (Rg) and the polydispersity (I = Mw/Mn) of the samples were determined using high performance size exclusion chromatography (HPSEC) combined with a multi-angle laser light scattering detector (MALLS). The system was composed of an HPLC system Prominence Shimadzu^™^, a PL aquagel-OH mixte, 8 µm (Varian) guard column (U 7.5 mm × L 50 mm), and a PL aquagel-OH mixte (Varian) separation column (U 7.5 mm × L 300 mm, operating range 10^2^-10^7^ g/mol). Elution was performed at 1 mL/min with 0.1 M ammonium acetate containing 0.03% (w/v) NaN_3,_ filtrated on 0.1 µm membrane (Durapore Membrane, PVDF, Hydrophilic type VVLP, Millipore). A differential refractive index (RI) detector (Hitachi L2490) and a multi-angle light scattering detector (Dawn Heleos II^™^, Wyatt) were coupled on-line and data computed with Astra software for absolute molar mass determination.

#### 3.3.3. Electrophoretic Mobility

Electrophoresis was performed in agarose gel: 0.7% (w/v) agarose gel was prepared in TAE buffer (0.04 M Tris acetate; 0.01 M EDTA, pH 8.5) and 30 µL of samples in native electrophoresis buffer (Bio-rad) were loaded and electrophoresis was run in TAE buffer for 2 h in a Maxi cuve (20 cm × 10 cm gel, Fisher Bioblock Scientific). Gels were fixed for 4 h in 25% (v/v) isopropanol and then colored over night by “Stains All” solution (1-Ethyl-2-[3-(1-ethylnaphtho[1,2-d]thiazolin-2-ylidene)-2-methylpropenyl]naphtho[1,2-d]thiazolium (Sigma)) prepared as follows: 5 mL of 0.001% (w/v) Stains All solution in dimethylformamide; 5 mL of 300 mM Tris-HCl pH 8.8; 5 mL of dimethylformamide; 25 mL of isopropanol, and 60 mL of H_2_O [48].

#### 3.3.4.Fourier Transform Infrared (FTIR) Analyses

Pellets were obtained by careful grinding of a mixture of 2 mg of polysaccharide with 200 mg of dry KBr. Infrared spectra were recorded with a Magna IR 550 Fourier Transform Infrared Spectrophotometer (Nicolet) equipped with a DTGS detector with a resolution of 4 cm^−1^. All the spectra were corrected from H_2_O and CO_2_ absorptions, using OMNIC software (Thermofischer Scientific).

#### 3.3.5. Microbiology

Sterilization efficiency was evaluated using a pour plate method. Every polysaccharide (0.2-0.4 g) was aseptically weighted, dissolved with 20 mL of sterile water and left at room temperature for 24 h for resuscitation and solubilization of polymers. The solutions were then serially diluted and 0.1 mL of each appropriate dilution was placed in an empty sterile Petri dish followed by the addition of molten agar tempered to 45 °C. For total flora (marine and non marine flora) 15 mL of PCA (Plate Count Agar) medium (Biokar Diagnostics, Beauvais France) was used. For marine flora, 15 mL of ZoBell medium (2216E medium) composed of sea-salts (Sigma, France), yeast extract and tryptone (Organotechnie, France) were added. After swirling and solidification of the agar plates, the latter were incubated aerobically for 48 h at 37 °C to develop colonies. Colonies were then counted and the data recorded. 

#### 3.3.6. Cytotoxicity of Ethylene Oxide Residues

To evaluate whether the level of ethylene oxide residue in treated polysaccharides was toxic, cellular viability of primary articular cartilage cells from rabbits (RAC) was measured using an MTS assay. RAC were isolated from five-week-old New Zealand white rabbits (Grimaud) as previously described by [49]. RAC were cultured in DMEM F-12 medium (Invitrogen corporation, France) supplemented with 10% (v/v) of fetal bovine (FBS) serum, 1% (w/v) of penicillin/streptomycin and 1% (w/v) of L-glutamine. RAC were allowed to attach in 24-well plates at a final density of 20,000 cells per well. After 24 h, the culture medium was removed and 1 mL of complete medium containing either HE800 EPS or GY785EPS at 100 µg/mL was added per well (corresponding to an amount of EO below 3 × 10^−3^ µg in 100 µg of EPS or <30 ppm). As positive and negative controls, RAC were also cultured in absence of polysaccharides or in the presence of actinomycin-D (5 µg/mL), an inhibitor of RNA polymerase, which was used as a potent inducer of cell death. Samples were incubated at 37 °C, 5% CO_2_. After 24 h, 48 h or 72 h of culture, culture media were removed. A Tetrazolium Salt (MTS) test (Promega, USA) to evaluate their mitochondrial activity was carried out. For the latter, a MTS solution was added in each well for 1 to 3 h according to the manufacturer’s instructions. The optical density of formazan dye was measured on a spectrophotometer at 490 nm. Each condition was tested in quadruplicate and the results were expressed as relative MTS activities as compared to the positive control.

#### 3.3.7. Statistical Analysis

Results are expressed as mean ± of quadruplicate determinations. Comparative studies of means were performed by using one-way ANOVA followed by post-hoc test (Fisher’s projected least significant difference) with a statistical significance at *P* < 0.05.

## 4. Conclusions

In our study, the results show that gamma or beta irradiation is completely unsuitable for the sterilization of the polysaccharides examined. For both EPSs, radiation induces great alterations in their physical structure with a loss of their rheological polymer properties and morphological structure, as demonstrated by a considerable decrease in their viscosity and molecular weight respectively. Surprisingly, radiation did not alter the chemical properties of the EPSs studied. The results obtained with cold plasma treatment were disappointing. This method is often described as a novel alternative for the sterilization of heat-sensitive substances. Our results showed that cold plasma treatment induces changes in both rheological and structural properties of the EPSs, with a decrease in their viscosity and molecular weight respectively. Our results also showed that the most adequate sterilization method for the sterilization of polysaccharides is EO treatment. This method provides both a good SAL and the lowest physical and chemical damage to the EPSs. The results obtained with EO are acceptable and compatible with subsequent use of these EPSs to investigate their therapeutic potential in animal models. Currently this treatment enables: (i) preservation of the non-Newtonian pseudo plastic behavior characteristic of both treated EPSs; and also (ii) retention of a high molecular weight close to the untreated EPSs. This study highlights the importance of the choice of the sterilization method, as an essential part of any product development.
